# Women’s experiences of homelessness and violence during the COVID-19 pandemic in Canada’s largest city: an integrated qualitative analysis of clients of violence against women organizations and encampment residents

**DOI:** 10.1186/s12889-025-22721-4

**Published:** 2025-04-28

**Authors:** Alexa R. Yakubovich, Lisa Boucher, Zoë Dodd, Alyssa Gerhardt, Priya Shastri

**Affiliations:** 1 Department of Community Health and Epidemiology, Dalhousie University, 5790 University Avenue, Halifax, B3H 1V Canada; 2 Nova Scotia Health, Halifax, Canada; 3 MAP Centre for Urban Health Solutions, Unity Health Toronto, Toronto, Canada; 4 Bruyére Health Research Institute, Ottawa, Canada; 5 Department of Sociology and Social Anthropology, Dalhousie University, Halifax, Canada; 6 Woman Abuse Council of Toronto, Toronto, Canada

**Keywords:** Violence, Homelessness, Women, Gender, COVID-19

## Abstract

**Background:**

Violence against women (VAW) is the most common cause of women’s homelessness. However, policy and programming for VAW and homelessness have developed and operated in siloes in many countries, including Canada, limiting capacity to address the unique needs of women facing both interrelated issues. This study uniquely analyzes data from participants experiencing violence and homelessness drawn from each of the VAW and homelessness sectors at the height of the COVID-19 pandemic in Canada’s largest city, Toronto.

**Methods:**

We qualitatively analyzed data from two studies conducted concurrently in 2021 as part of the Marginalization and COVID-19 (MARCO) Project, which aimed to investigate outcomes of the COVID-19 response among people experiencing marginalization in the Greater Toronto Area. Participants were 10 survivors who accessed VAW services and 23 residents of homeless encampments. We applied a reflexive thematic analysis within a feminist poststructuralist framework to explore participants’ experiences of violence and homelessness and interrogate the structural factors that dictate which and how different participants ‘end up’ in different sectors and their outcomes.

**Results:**

We generated three themes in our analysis: (1) inequities exacerbated: (abuse of) power and control as pathways into women’s homelessness; (2) negotiating trade-offs between safety and autonomy; and (3) gender stereotypes versus gender-transformative approaches. There was a common pathway of VAW to homelessness, exacerbated by the COVID-19 pandemic and most acutely felt by participants facing intersecting forms of marginalization (e.g., economic or immigration precarity). Considerations around safety and autonomy were central to shaping experiences of women’s homelessness. Participants described ways in which gender stereotypes, both internalized and at systems- and organizational-levels, harmed them in terms of service (in)access – especially for women who used substances or were lone caregivers. The most positive experiences when accessing shelter or housing services were when participants received gender-specific supports that promoted their safety and empowerment – in many cases, in spite of the constraints presented by COVID-19 public health measures.

**Conclusions:**

Our results highlight the need for strengthened collaboration between the VAW and housing/homelessness sectors and a unified policy strategy to address homelessness that applies a gender-transformative and intersectional approach, during and beyond public health emergencies.

**Supplementary Information:**

The online version contains supplementary material available at 10.1186/s12889-025-22721-4.

## Introduction

Gender-based violence is violence or abuse (most commonly intimate partner violence and sexual violence) committed because of a person’s gender or which disproportionately affects people of a certain gender [1–3]. The most common victims of this violence are women, while gender expansive people are also disproportionately affected [4–6]. Gender-based violence against women (VAW) is both the leading cause and a common outcome of women’s homelessness [7]. VAW increased during the COVID-19 pandemic due to stressors like physical distancing and isolation, income loss, precarious employment, and service disruptions [8, 9]. At the same time, homelessness also increased, due to rising costs of living and economic precarity, an inadequate supply of affordable housing, limited incentives for affordable housing builds, and insufficient supportive housing services [10–12]. The rising burdens of VAW and women’s homelessness are linked and inequitably experienced by women facing intersecting forms of marginalization, including racism, sexism, homophobia, transphobia, ableism, or poverty [13–15]. In turn, both VAW and homelessness have serious consequences for women’s health, including injury, mental health problems, and chronic disease and pain [16, 17]. As a result, preventing VAW and women’s homelessness has been recognized internationally as a public health priority [18, 19].

Despite the inextricable links between VAW and women’s homelessness, in Canada and many other countries worldwide, each of these issues has typically been addressed by separate services and policies, with little to no systematic coordination [15]. In part, this is due to the failure of routine data collection on homelessness – which typically involves point-in-time counts of the number of people living in homeless shelters or on the streets – to account for the most common ways that women experience homelessness [12]. Women facing VAW-related homelessness instead often rely on accommodation that is provisional (e.g., someone else’s home), overcrowded, unaffordable (i.e., where they have difficulty meeting basic needs), or remain living with violent partners because they cannot afford to leave or fear that their child custody will be impacted if they do [13, 20]. Homelessness prevalence data thus more commonly reflect the experiences of men (especially heterosexual, cisgender men), whereas experiences of homelessness among women and gender expansive people tend to remain ‘hidden.’

The hidden nature of women’s homelessness has reinforced the siloed funding and work of the VAW and homeless service systems and contributed to homelessness policies, programs, and evaluations that lack a gendered lens and do not adequately address the unique needs of women or gender expansive people experiencing violence and homelessness [21]. As we have outlined in previous work, this includes Canada’s National Housing Strategy, which prioritizes the prevention of ‘chronic homelessness’ (i.e., currently homeless and have spent 180 cumulative nights in emergency shelter, unsheltered locations, or temporary housing), for which women experiencing hidden homelessness often fail to meet the definition [15, 22]. In some other contexts (e.g., the UK and USA), rapid rehousing models (including Canada’s most popular program, Housing First) and other long-term supportive housing models have been adapted to incorporate a gender transformative lens with the inclusion of VAW-specific wraparound supports [23]. However, few implementation examples let al.one evaluations of these gender-based adaptations exist in Canada [15, 18]. As a result, despite the intertwinement of VAW and gender-based homelessness, there remain two largely separate systems of policy and practice designed to address each issue in isolation, with little coordination and, critically, investment that would foster that collaboration [15, 24].

This article represents a unique opportunity to analyze data from participants experiencing violence and homelessness drawn from both the VAW sector and homeless encampments at the height of the COVID-19 pandemic in Canada’s largest city, Toronto. Using a feminist poststructuralist framework [25], we aimed to critically examine how women’s experiences of homelessness and access to different housing and sheltering options were socially constructed and impacted by power relations. Our ultimate goal is to inform the increasingly recognized need for intersectoral collaboration and strengthened policy responses to homelessness and violence among women [15, 26] by interrogating the structural factors that dictate which women end up engaging in the homelessness or VAW sectors and the ways in which commonalities in their experiences can be better served by coordinated programming and policy across systems.

## Methods

We used data from two studies conducted concurrently in 2021 as part of the Marginalization and COVID-19 (MARCO) Project, which aimed to investigate the experiences and outcomes of the COVID-19 response among people experiencing marginalization in the Greater Toronto Area (GTA). Investigators from all MARCO Project studies met regularly throughout the study process (including design and data collection) to discuss common methods and opportunities for shared aims and outputs. The MARCO-VAW Study examined the process, experiences, and outcomes of adapting VAW programming in the GTA during the height of the COVID-19 pandemic [24] and the MARCO-Encampments Study examined the experiences of the increased number of encampment residents and workers or volunteers providing outreach supports to them [27]. The two studies applied a transformative paradigm, which aimed to centre the experiences of people experiencing marginalization and promote social and structural changes that reduce inequities [28]. Both studies were community-partnered and used mixed-methods. The current analysis relies on qualitative data collected from the semi-structured interviews with VAW clients (MARCO-VAW Study) and encampment residents (MARCO-Encampments Study). Participants in both the MARCO-VAW and MARCO-Encampments studies provided written informed consent prior to their interviews and were offered a $40 honorarium. Interviewers from both studies received the same foundational training on anti-racist and anti-oppressive practices via the MARCO Project, with supplementary training by each study team (e.g., on VAW research methods for the MARCO-VAW interviewers). Research staff provided participants with contact information for local supportive resources post-interview as needed. Ethics approval was provided by the Unity Health Toronto (REB#20–124), Dalhousie University (REB#2022–6275), and University of Ottawa (REB#H-03-21-6715) Research Ethics Boards. Full information on study methods is available online [29, 30] and in previous articles [24, 27]. The following sub-sections briefly summarize the methods used in each study, followed by our analysis strategy. Interview guides are included in Additional file 1.

### The MARCO-VAW Study

The MARCO-VAW Study was led by an academic researcher and community-based researcher with a team of academics, peer researchers (women with lived experience of violence who received training on VAW research methods), and trainees as well as an advisory group representing 42 VAW organizations across the GTA. The study’s co-leads and peer researchers conducted interviews (often in pairs) from April to September 2021 with VAW survivors who had accessed supportive services during the COVID-19 pandemic (since March 2020) in the GTA. Participants were purposively recruited with the support of collaborating organizations and networks with the goal of maximum variation in socio-demographic characteristics and services accessed [31]. Participation was open to anyone within an inclusive definition of ‘women’ (including trans or cisgender) or nonbinary gender identities, with any sexual identity, in line with the most inclusive service eligibility criteria of VAW organizations. Interviews were conducted with interpretation services in three instances in participants’ native languages. At the end of the interview, participants completed a short socio-demographic survey with the interviewer. Interviews were conducted virtually and transcribed verbatim. Interviews were semi-structured and covered five main topics: about you, impact of the pandemic, experiences of violence, service access and outcomes, and contextual factors (experiences of discrimination and recommendations for policy). Interviews ranged from 90 to 150 min and tended to be narrative in nature, with survivors describing how their life histories led to and intersected with their pandemic experiences. Participants’ experiences of housing and homelessness were actively explored throughout interviews.

### The MARCO-Encampments Study

The MARCO-Encampments Study was co-led by a PhD candidate, MD-PhD student, and Master’s-educated community researcher with lived experience; all of whom had extensive experience working with marginalized groups in prior community-based research or service provision. Participants were recruited from six Toronto-based encampments, chosen because of their prominence in the downtown area, large sizes, proximity to various community services, and contextual characteristics (e.g., residents with diverse or similar identity factors). Current or former encampment residents, recruited through convenience and snowball sampling with the support of community groups providing outreach at the six sites, first participated in a survey. Following this, interview participants were purposively sampled based on responses to the survey questions (especially demographics) to maximize variation [31]. Interviews were conducted one-on-one, in English, and in-person within the encampments in locations where conversations could not be overheard. Three research staff, including a researcher with lived experience, conducted interviews between March-June 2021. Whenever possible, participants were paired with an interviewer who shared some aspect of their identity (e.g., Indigeneity). Interviews were audio-recorded and transcribed. The semi-structured interview guide explored wide-ranging experiences among encampment residents: encampment living and interactions with other residents and those external to encampments; COVID-19, health, and substance use; outreach supports; and shelter or housing supports. Interviews ranged from 11 to 86 min.

### Analysis

We used a reflexive thematic analysis within a feminist poststructuralist framework [25, 32–34]. The MARCO-VAW and MARCO-Encampments datasets were first each collaboratively coded by a team of researchers. Team members familiarized themselves with the data, took notes on their observations, and met regularly to discuss their perspectives. They then developed codes to summarize salient features of the dataset relevant to each origin study and met to discuss, refine, and integrate different perspectives into the coding. Next, we created a combined dataset with data from each study relevant to women’s experiences of homelessness and violence based on these codes. This included all data from the MARCO-VAW Study related to participants’ experiences of homelessness or shelter (including both VAW or homeless shelters). In line with MARCO-VAW, from MARCO-Encampments we sought to include any data on experiences of violence, homelessness, or shelter from participants who identified as women (cis or trans) or nonbinary as well as data from participants who identified as men but spoke about VAW or women’s homelessness (e.g., promoting women’s safety in encampments), or gender-based violence or homelessness more broadly. We aimed to extend previous applications of feminist poststructuralism to women’s homelessness research [35] and identify ways in which participants’ descriptions of their experiences demonstrate power relations and social discourses around gender, violence, safety, and homelessness [25]. We examined how these discourses affected women at the intersection of other personal and social factors, and, in turn, ways in which policy and systems of care can be more equitably restructured. We generated themes based on patterns observed in the combined dataset, paying particular attention to areas of divergence and convergence between the MARCO-VAW and MARCO-Encampments data. Team members met regularly to discuss and make revisions until we agreed that the framework most robustly captured the meanings we identified across the dataset with theoretical and practical implications for social and structural change.

A variety of strategies were employed throughout data collection and analysis to increase quality and rigour in both studies [36–38]. These strategies included: investing time in rapport-building with participants; conducting semi-structured interviews that allowed participants to expand on experiences as they came up in conversation; making field notes and memos throughout data collection and analysis to collect rich details on participant encounters and document study decisions and the development of our findings; using thick description (e.g., reporting relevant background information and lengthier quotes) to facilitate readers’ capacity to determine meaningfulness; including diverse members in our team and decision-making to intentionally incorporate a variety of perspectives into our interpretations; and discussing our interpretations with broader knowledge users (including advisory group and sector-wide feedback sessions) to further refine, contextualize, and enrich our conclusions. We have selected quotations to support our analysis and included anonymized participant identification numbers to locate and demonstrate the scope of the data used [39]. MARCO-VAW and -Encampment participants can be distinguished by the starting letter of V or E in their identification numbers, respectively, and we provide relevant sociodemographic information to help ‘locate’ exemplar quotes.

## Results

Ten participants were interviewed for the MARCO-VAW Study and 23 were interviewed for the MARCO-Encampments Study. Table 1 summarizes the sociodemographic characteristics of all 33 participants by origin study. One notable difference was that most MARCO-VAW participants were cisgender women (90% versus 39% for MARCO-Encampments); however, across both studies, most data focused on gender-based violence or homelessness came from cis women participants. MARCO-VAW participants were also about 10 years older on average and tended to have more education than MARCO-Encampments participants. Both participant groups were racially diverse, but 35% of MARCO-Encampments participants identified as Indigenous while none of the MARCO-VAW participants did. Both sets of participants were facing significant economic disadvantage at time of interview: most MARCO-Encampments participants were receiving disability or social assistance payments, while 90% of MARCO-VAW participants had a household income less than $20,000.

**Table 1:** Sociodemographic characteristics of the analytic sample by origin study

Sociodemographic characteristic	MARCO-VAW (*N* = 10)	MARCO-Encampments (*N* = 23)
**Gender** ^**a**^		
Cisgender woman	9 (90%)	9 (39%)
Cisgender man	0	11 (48%)
Gender expansive or transgender^b^	1 (10%)	2 (9%)
**Sexual identity** ^**a**^		
Heterosexual or straight	8 (80%)	14 (61%)
Lesbian, gay, bisexual, queer, or other	2 (20%)	8 (35%)
**Race**		
White	3 (30%)	9 (39%)
Black	2 (20%)	4 (17%)
Latin American	2 (20%)	0
South or East Asian	3 (30%)	1 (4%)
Middle Eastern	0	1 (4%)
Indigenous	0	8 (35%)
**Age**,** Median (IQR)**	47 (40–56)	37 (30–50)
**Education** ^**a**^		
Less than high school	0	9 (39%)
High school	1 (10%)	5 (22%)
Some college or university	0	3 (13%)
College or university certificate/diploma	9 (90%)	5 (22%)

^a^One participant from the MARCO-Encampments Study did not provide data on their gender, sexual identity, or education, hence totals do not add to 100% for these variables

^b^Participants in this category identified as either transgender women or nonbinary (e.g., gender fluid)

MARCO-VAW participants had accessed several different types of VAW programming during the pandemic, including: mental health or crisis support (100%), shelter (70%), children’s aid (60%), healthcare (50%), second-stage housing (40%), and legal advocacy and support (40%). 65% of MARCO-Encampments participants were living in encampments at the time of interview, while 35% were former residents (having moved into shelter or housing at time of interview but lived in encampments during the COVID-19 pandemic). Duration of living in encampments ranged from several weeks to over one year.

We generated three themes to understand women’s experiences of homelessness during the COVID-19 pandemic: (1) inequities exacerbated: (abuse of) power and control as pathways into women’s homelessness and the impact of the COVID-19 pandemic; (2) negotiating trade-offs between safety and autonomy; and (3) gender stereotypes versus gender-transformative approaches.

### Inequities exacerbated: (abuse of) power and control as pathways into women’s homelessness and the impact of the COVID-19 pandemic

There was a clear pattern across participants’ stories in the ways in which gendered differentials in power and control created unsafe situations of (typically hidden) homelessness, demonstrating the common pathway of violence to homelessness among women across the VAW and housing and homelessness sectors. For instance, one encampment resident (E21, white woman, age 29) shared, “I was staying with my uncle […] He’s super, super controlling, super kind of like, emotionally, a little physically […] and like, he wouldn’t let me go out. Like, I have to come home with my methadone receipt, to prove that I’m on methadone, shit like that. […] So, I’m like, ‘Yeah, fuck you. I’m outta here.‘” Another resident (E14, Indigenous woman, age 32) shared,

Okay, so yeah, so I was in a bad relationship. I decided to run away from the relationship. Got a condo […] My ex decided that he wanted to get me kicked out of the condo, because he wanted me to be where he was, which was on the street and being a loser. […] He went to the condo, beating on everything, on the floor, on every floor. And then he went and sexually, like, not sexually assaulted, but like, made vulgar comments to the lady at the front desk. […] And yeah, so they put us both out. But he got arrested and I, yeah, had to leave, I guess.

These excerpts illustrate some of the different ways in which men (often with greater social and economic power) leveraged control over women in their lives, including through physical, sexual, and psychological forms of abuse, ultimately forcing them into homelessness. This included sabotaging housing options in the context of limited affordable or supportive housing available to meet women’s complex needs. Oftentimes, women experienced this violence from partners, ex-partners, or family members, but participants across both samples also described instances of VAW from neighbours, landlords, and even program and city staff that affected their living situations and ultimately experiences of homelessness. When participants made the decision to leave abusive situations, they were commonly left with limited or no access to their belongings and socioeconomic resources.

In most cases, including both encampment participants above, participants had longstanding histories of trauma, abuse, and homelessness that preceded the COVID-19 pandemic. However, the pandemic impacted participants’ experiences of abuse and homelessness in several ways. First, both VAW and homeless services were overwhelmed by COVID-19, with their capacity further limited by public health mandates in the first waves of the pandemic, making it more difficult for women to escape their living situations. For instance, one VAW participant (V74, white woman, age 52) shared:

I was getting $1000 from Ontario Works [social assistance] in Toronto, and that had to pay for everything. So anything over 800 I couldn’t. And I have seen a lot of places and they are terrible, as you can probably imagine. So, I do want to say that there was a plan to go [rent our neighbour’s basement] and then COVID came, quashed all of that. I started calling all the time to see what was available in shelters because I wasn’t sure. […] I’m calling every day. And the search went from Toronto to, I’m looking, I’m talking to people […] everywhere in southern Ontario. I have no car, but I can’t let that stop me. And there’s nothing available. So, we stay.

This participant repeatedly exercised her agency to challenge the structural barriers that kept her and her daughter from safe housing, ultimately to no avail for months – demonstrating how rising costs of living and reductions in shelter spaces (including shelter hotels) during the first waves of the pandemic were most acutely felt by those experiencing economic precarity, without an adequate social safety net. This was a commonality across VAW survivor and encampment resident participants. The risk and experience of women’s homelessness were further exacerbated for participants facing multiple forms of marginalization (e.g., histories of incarceration, substance use, precarious migrant status, mental health problems, or disability), which additionally limited options for safe and affordable housing.

For some women seeking support, capacity limitations resulting from public health measures meant that only emergency homelessness (not women’s) shelters were available to them. One VAW survivor (V73, black woman, age 27) described her inability to access a women’s shelter with her children after leaving an abusive situation:

It was super tough during the pandemic. I actually, I used to live with my mom. She was my abuser and so I lived through it since I was 17 years until now. But, during the pandemic, I had to move out since September with my two autistic girls. […] Because she almost hit me in front of my kids, and I had no choice. And so therefore I had to move, and I moved to emergency shelter […] They told me the women’s shelters are like full capacity, like I had no chance in the world because how many women could even go in there? […] If there was 12 rooms available before the pandemic, now […] there’s only six bathrooms, only six people could live there. Yeah, so, it really put down my chances of getting a woman’s shelter ASAP, so I could’ve got that support. So, they just took me to [what] the best bet was.

As this quote clearly demonstrates, participants faced a system that offered them little choice as they sought safety and security. For many participants, like V73, who did not receive gender-specific supports, the resulting experience was retraumatizing, as discussed further below. Relatedly, the pandemic also led to many participants being forced into prolonged sheltering in place with violent partners or family members. As a result, in some cases, COVID-19 as an infectious disease was exploited within the coercive and controlling dynamics of abusive relationships. For instance, one VAW survivor (V78, Latina woman, age 36) shared:

I think that it was hard for him to get a job, right? […] So, he was a stress. The lease of the house where we were living, it was going to end and we were looking for another place, but they were very expensive. So, the baby in the house, we sharing the space all the time. All the time because everything was closed. I couldn’t go to a mall because I had to be there, because it was a lockdown. So, it was all the time, no job, you know, like he was drinking. […] And you know what? He’s one of these persons that they don’t believe in COVID. They don’t believe that is true. It’s all a lie for the government, it’s, you know, so he was like, ‘I’m not going to wear a mask. It’s against my beliefs.’ […] So, it was all of this stuff because we were fighting about that, right? Like, ‘You have to take care. You have to, you know, like, you have to think about us.’

This participant’s experience of abuse highlights the myriad ways the pandemic exacerbated VAW due to stressors around job loss, housing precarity, increased substance use, and physical and social isolation. In this case, the participant’s partner flaunted COVID-19 restrictions, heightening her fear and disempowerment within the relationship, while intersecting social factors (e.g., a new baby, economic precarity) exacerbated their power imbalance. In addition to space limitations, sheltering in place in abusive situations during the pandemic was another challenge to navigating women’s homelessness as participants had little opportunity to plan and prepare to leave without their abuser’s awareness. These situations posed significant risks to women’s safety given that the time immediately following separation is one of the highest risk periods for intimate partner homicide [40].

### Negotiating trade-offs between safety and autonomy

Beliefs, values, and practices around safety and autonomy were key factors that shaped participants’ decisions around their (typically limited) living options and experiences of homelessness, often in complex ways. Most MARCO-VAW participants who accessed shelter accessed VAW shelters; one participant accessed only an emergency homeless shelter (V75) and one participant accessed both emergency and VAW shelters (V73). In addition, two participants (V72 and V77) accessed two different VAW shelters, offering unique perspectives on the different supports available, as discussed further below. In contrast, MARCO-Encampment participants exclusively accessed homeless shelters (never VAW shelters), with two participants accessing women’s homeless shelters (E12 and E22). There was a common pattern amongst VAW and encampment participants that their preferred living options struck the best available balance of safety versus autonomy, or that this balance influenced their experiences of living situations. However, data from encampment participants tended to be more focused around the dichotomy between emergency homeless shelter versus encampment living, by virtue of the living options that were (un)available to them.

#### Social control and physical barriers in emergency homeless shelters

In describing the shelter system, and especially emergency homeless shelters, participants spoke of a lack of personal autonomy and social isolation caused by the rigidity of shelter rules (around e.g., staying in rooms, curfews, not being allowed outside, eating arrangements), which became even stricter due to pandemic restrictions. Participants used words like “prison,” “trapped,” “stuck,” “restrictive,” “invasive,” and even “hell” to describe their time in shelter, which encampment participants then typically contrasted with the self-governance of encampment living. Participants often interpreted shelter rules as arbitrary and belittling, creating an environment of heavy surveillance that was unsettling for residents. For instance, one encampment participant (E14, Indigenous woman, age 32) shared:

They don’t treat us like anything but like we’re their job. Or, like, we’re just entertainment. Like, we honestly think that we’re, like, on Big Brother. […] We see cameras everywhere. Like no wonder we’re fucking all going crazy. Because these people are antagonizing us with this shit. […] They want to distract us from getting where we have to go, getting an apartment. Instead, we’re too busy fighting staff to have a drink in our room. Like, we’re not fucking children.

This quote illustrates the social control the shelter exerted over residents – suggesting an underlying systems-level assumption that disorder would otherwise prevail – and the ways in which that in turn disempowered residents and, as this participant describes, impeded their progress in exiting homelessness. This quote further demonstrates a few common patterns amongst participants’ stories: first, that in many cases, shelters’ rules and physical environments exacerbated safety risks for residents and, second, that it was not only other residents who threatened women’s safety and wellbeing but staff themselves. This was especially true for women living with disabilities or who used substances. For instance, one encampment participant (E23, white woman, age 50) living with a physical disability shared the following on her time in a homeless shelter operating out of a hotel:

They refused to move me [from a high floor to a lower floor] […] denied access to the wheelchair ramp, because that door was exit only. […] And three months later, there was a fire where they evacuated the rest of the building, and they came up and said if I couldn’t do the stairs, there’s nothing they could do, and they left me behind. And I thought I was gonna die. And I spoke to other disabled people in the building, and all of us that couldn’t go down the stairs were left behind.

This participant went on to contrast her traumatic experience in a homeless shelter with living in an encampment: “Living in the park was the first place where […] I actually started feeling like me. […] And because I’d never lived anywhere accessible and had, like the chair, I couldn’t, like – I love nature and parks, but I can’t get to them.” This participant’s story demonstrates the inaccessibility of the shelter’s physical environment (and indeed any other living option she previously accessed). It also demonstrates the inadequacy of emergency preparedness planning and strict adherence of staff to shelter rules that fell short of meeting participants’ needs – likely resulting from insufficient training and resourcing. The outcome in this case was that this participant’s first experience of (and possibly only available) accessible living was in an encampment.

The physical environment of shelters, combined with limits on personal autonomy and social isolation resulting from shelter rules, led to many participants feeling as though they were fending for themselves in the shelter system, compared to encampments. For example, participants discussed fearing or experiencing having their personal space violated or possessions stolen by both residents and staff. Moreover, several participants talked about their own experiences of or witnessing VAW in emergency homeless shelters, without staff intervention. For instance, one encampment participant shared (E14), “My boyfriend was fucking, was beating me up on the other side of the door of the office, and they refused to come see me or help me. […] They said they were afraid for their own lives, that they didn’t want to step in. And left me there.” This excerpt demonstrates the lack of capacity to respond to VAW within the homeless shelter system and the detrimental impacts on women’s safety and well-being. Indeed, one VAW survivor participant (V73) contrasted her experiences in an emergency homeless shelter:

We need to go to a women’s shelter […] because the emergency shelters are horrible, like, literally, like, I was feeling very depressed. […] It wasn’t clean or anything. […] We was hearing other families. People could have brought alcohol, like, you would have heard people yelling, we felt very unsafe there. My kids would literally sleep right on my chest, both of them.

With her experiences in a VAW shelter:

The [VAW] shelter was a lot better. It was clean. […] Staff was always there 24/7. The emergency shelter where I was in [month], staff wasn’t there 24/7. They would check in with you, but they didn’t care about your story. […] [In the VAW shelter] if [my kids] fall asleep, I wasn’t scared to go downstairs if I needed to talk to the staff. But [in the emergency shelter], I couldn’t do that because I had nobody else to talk to.

As this participant demonstrates, for women fleeing violent and abusive situations, especially those with children in their care, being further subjected to violence and abuse in homeless shelters was traumatizing. The lack of gender and VAW-specific supports, including social support, further compounded these traumatic experiences.

#### Autonomy and safety in encampments

Encampment participants commonly connected heightened safety concerns for people who use substances to the relative isolation of shelters compared to encampments. Several participants noted they knew or had heard of someone who had died in the shelter system by overdose or otherwise suspicious circumstances. For instance, one resident shared (E21, white woman, age 29): “I also know, like, now three people who have passed away in these [shelter] hotels. Cause they don’t know how to reverse an overdose. […] You shouldn’t be able to work at a shelter where you don’t know how to reverse an overdose.” As was the case here, the lack of a harm reduction approach in shelters was another reason underlying participants’ distrust of shelter staff, which combined with individualistic and surveilled living, often made them consider encampments as safer places to use substances. For instance, one encampment participant explained (E12, Indigenous, previously stayed at a women’s homeless shelter): “Honestly, as an opiate user, I think it’s a lot safer [to use in an encampment compared to shelter] cause there’s always people around to make sure you’re okay. […] We usually try and, like, at least sit together or, like, you know, you’ll say to somebody, like, ‘Hey just come back and check on me in five minutes,’ kind of thing.” As this participant demonstrates, without the rules of shelter, encampment residents commonly felt that they could exercise their agency and create their own regulatory systems that were informed by their lived expertise and met their needs.

More broadly, greater autonomy in encampments allowed some participants to benefit from networks of mutual support and protection not possible within shelter environments, as described in an earlier MARCO-Encampments analysis [27]. Notably, however, this often involved participants relying on male protection in the encampments. For instance, one encampment participant (E20, white woman, age 31) described:

Other encampments, I’ve heard, like, it’s not even, like, it’s not safe for people really to be there, especially, like, females to be there. Here, like, I know most of the guys around here, so this is pretty, this is pretty okay. So, if I wasn’t right here, if I wasn’t in this situation, then I probably wouldn’t say it was a pleasant experience.

Participants recognized that there were safety risks, particularly for women, across homeless shelters and encampments (including multiple participants who discussed rape and other forms of sexual assault). However, they commonly discussed preferring encampment or rough living for the sake of greater personal autonomy, especially in terms of protecting themselves. In contrast to homeless shelters, where participants often felt personally, or that women in general were, vulnerable to violence without recourse, encampments allowed participants to define their own rules and consequences for rule violations. In some cases, this entailed intimidation and physical violence, typically by men (e.g., male encampment participant, E9: “You put your hands on a woman out here. You’re going to get it. I’m not calling the police. We’re going to beat you.”). However, two female encampment participants (E14 and E23) also described taking on ‘motherly’ roles in the encampment as a way of promoting safety and security. This involved building relationships with other residents, ensuring their needs were met, and, in one case (E23), mediating and at times ‘disciplining’ others to resolve conflicts (e.g., “using my mom voice or […] pretending that I was angry, or disappointed in them, and yelling”). Participants also described carrying weapons or pepper spray as strategies to keep safe in encampments.

As in shelters, participants described situations where staff, not just other residents, contributed to the harm they experienced in encampments – in some cases, as serious as perpetrating VAW. For instance, one gender non-conforming encampment participant who identified as lesbian/gay and a mother (E10) shared:

Most of this staff are good. I have a picture of one of the men on there. Yeah, I think he was just stalking me. I took a picture of him, standing right there looking at me and he’s tried to have a couple of conversations with me in there [the nearby recreation centre]. […] And then I actually said something to him […] and I went out the door and he comes out the door after me and yells, ‘Fuck off.’ This is a city staff. […] I feel I should make a formal complaint to the city about that guy’s behaviour. […] You make a complaint, you get evicted, you make a complaint, you get kicked out. […] Every camper in every park is dealing with that all the time. We’re the unwanted members of society. And at any given time, we can all be criminalized, you know, and kicked out of the park.

As the excerpt illustrates, this participant felt deeply uncomfortable by the behaviour of a staff member, who, at best, failed to appreciate the gendered power differential at play. The participant expresses a social discourse around the criminalization of homelessness, and how they exercised agency by standing up to the inappropriate behaviour, while also acknowledging that engaging the broader system would be unlikely to work in their favour. Another participant (E3, white woman, age 46) described moving her tent closer to where police were regularly present as a way to keep safe before realizing, “I was worried about other people. But then afterwards I became aware that the police became the harassers.” This participant likewise evoked the criminalization of homelessness, discussing how she sought out police protection as a “single female” but then realized the police were “looking for drug use or looking for whatever reason, for any excuse, to pick up people.” Taken together, participants’ stories of interactions with homeless shelters and people in positions of authority demonstrated numerous ways in which participants felt disempowered and often, as a result, felt encampment or rough living provided opportunities to live autonomously.

#### Promoting human dignity

When women preferred shelters, it usually involved considerations around enhancing human dignity. For instance, one encampment participant (E22, black woman, age 49) described splitting her time between a women’s homeless shelter and an encampment to draw on the benefits of each, explaining:

I’m half in the encampment, and half in women’s res, just because it’s easier to stay clean when you have access to, you know, their washers, their showers. I can wash my clothes easier. […] And I don’t have to be in women’s res until 3:30. So, it was, like, you know, a gift. It was hard to give up the whole shelter thing, but the food is way better out in the encampment, I can say. And it gave me the opportunity to realize that humanity really does care about each other.

The participant’s use of both settings allowed her to maintain a balance of autonomy, social support, and safety. Notably, she was able to achieve this in a women’s shelter; several VAW participants also described positive, and in some cases transformative, experiences in VAW shelters, which we discuss in the final theme. Two encampment participants were also given a tiny shelter (small, insulated, and temporary single-dwelling units) to live in, which similarly was experienced as empowering in its balance of safety and autonomy.

The above excerpt further illustrates two components related to human dignity that were common among participants’ appraisals of their living conditions: personal hygiene and food. Participants very much valued when they had safe access to showers and washrooms, whether in shelters or encampments. Likewise, participants’ negative or positive descriptions of their living conditions often centred around the nature of their access to food and its quality, which is reflected in a more detailed analysis of food insecurity among MARCO-VAW participants [41]. For instance, one VAW survivor participant (V75, black woman, age 46), in discussing her time in a homeless shelter, shared: “I do wish that they kind of had -- I’m not going to say a menu because, I mean, they’re not catering to homeless people, but at least have, like, options.” As both this quote and the above excerpts demonstrate, there was a common conception among participants that shelter food was poor quality. The lack of choice over food in shelter was yet another means by which participants felt disempowered in their experiences in both VAW and homeless shelters. For instance, one VAW participant (V77, South Asian woman, age 44) connected her anemia to the lack of nutritious food available in the first VAW shelter she stayed at and lamented: “In shelters, there was no food for me. Because of COVID, there was restrictions. I couldn’t cook in the kitchen. I can’t go out to buy my food.” In contrast, at the second VAW shelter she stayed at, “They were feeling like home, that that shelter was not like prison, you can go in the kitchen, the cooks are good, they behaved good with you.” Having personal control and autonomy over food was one way in which participants were able to reclaim power while experiencing homelessness. When this did not happen, the experience sometimes served as an extension of the controlling environments participants had fled from.

### Gender stereotypes versus gender transformative approaches

The social construction of gender played a central role in the services participants accessed – both in terms of the supports that were available to them and what they felt they could or deserved to access – and how well services met their needs.

#### ‘Deserving’ women

Several participants emphasized why they were or were not deserving of the support they had needed or received since becoming homeless. In this context, support was seen as something that needed to be earned and there were certain characteristics that made some women deserving of this support and others not. For example, one VAW participant (V75, black woman, age 36) distinguished herself from other shelter residents to explain why she thought she received preferential treatment from shelter staff:

The staff saw something different in me. I’ll be honest, I think I got some preferential treatment. […] They knew that I was educated. They knew that I was, like, I was going to church on a weekly basis. They knew I was apartment hunting. If I had nothing to do, I was going to the library to go on the computer and try and find something to do. All the resources that they offered, like I was just taking it. And so, like, they really liked me […] When you’re at the shelter, I’ll see maybe, like, 85% of the, I guess, the people that are there, like the homeless people, they’re happy in that situation. Like, they don’t have any plans to go anywhere. They don’t pay rent. Their money is used on drugs. And so, they look forward to their O.W. [social assistance] at the end of the month and within a week it’s gone. Like they don’t really look forward to anything else.

This participant highlighted specific attributes that she viewed as positive about herself—looking for work, churchgoing, educated—in contrast to the traits she saw in others living in the shelter (e.g., substance use) who staff treated differently from her. This excerpt demonstrates the social discourse around the ‘undeserving poor,’ wherein experiences of homelessness or other forms of poverty are understood as individual-level problems requiring individual-level solutions (e.g., work harder, stay abstinent). An encampment participant (E22, black woman, age 49) expressed a similar sentiment, instead emphasizing why she did not receive support from women’s transitional housing staff: “Well, they basically told me, yeah, you know, they didn’t want me to stay there. Because this is for women who are trying to change their lives. And you know, trying to make it, structure, and to be out looking for a job, and, you know, to be out doing something with yourself. And that’s not looking for drugs and then sleeping all the next day.” This participant demonstrates the gendered nature of the discourse on the undeserving poor (e.g., it is especially reprehensible for women to use drugs), which creates additional barriers for women experiencing homelessness and violence to access gender-specific supports. This is notable given the total lack of encampment participants – who made up the majority of women who were using substances in this sample – who accessed any VAW-specific supports.

In addition to substance use, newcomer participants demonstrated the additional guilt that gender-based stereotypes around poverty and social assistance created for them. For instance, one VAW survivor participant (V78, Latina woman, age 36, newcomer) shared:

And sometimes, I feel, like, bad, you know, like I feel like what I’m doing here, you know? Like, I should be working, and I should be doing something, you know, like for me and for the baby. […] I’m very thankful to be here because sometimes I feel like I don’t deserve it, right? Because I have this in my mind, like I’m not Canadian. What I’m doing here, they are helping for free, right, like, I think that I am in debt with them. So, I’m very thankful to be here just to have a room with my baby, to have food in the fridge, right? So, I don’t want to ask for more, you know?

This participant was staying in a VAW shelter without any childcare support during the pandemic – she described having to hold her baby at all hours while trying to complete the paperwork to secure her residency status under humanitarian and compassionate grounds. Yet, she did not feel deserving of any more support because of her precarious migrant status – illustrating how the social discourse on the undeserving poor includes undocumented migrants, marginalizing this participant further as a single caregiver.

#### The intersection of homelessness and motherhood

Most VAW survivor participants had dependent children living with them. In contrast, only three women encampment participants described having children (one of whom had an adult-aged child while the remaining two did not have child custody). This is notable in and of itself in terms of understanding *which* women experiencing homelessness ended up accessing VAW versus general homelessness supports and the suitability of those supports.

For VAW participants living with children, experiences of shelter life centred around whether they felt they had the agency to care for their children as they would like and supports available to meet their children’s needs. For instance, one VAW survivor (V73, black woman, single mother) described the efforts she made to ensure her children could attend online schooling during the pandemic, despite having limited internet access in an emergency homeless shelter:

I used to drive to Tim Horton’s, grab their breakfast, grab myself a coffee, go sit right in the middle between my kids and sign up to the Wi-Fi there and do schooling in my car. They [the emergency shelter] had no Wi-Fi, and the only way you get Wi-Fi, it’s going literally in the main, you’ve got to go out of your room, go downstairs, and that’s where everyone else was. And they had some COVID positives there. And I didn’t feel comfortable knowing my kids wasn’t wearing a mask. So, like, as soon as we used to wake up, we got dressed. When we’re leaving, we’re literally leaving for the whole day. Like I didn’t care how much money. Thank God for savings because that place didn’t help me with no money, no nothing. And so, like, I got my savings out and I had to spend throughout the whole month, gas, money, everything. It was really a rollercoaster.

This excerpt demonstrates the psychological stress and socioeconomic impacts on single caregivers when homelessness supports did not meet their family’s needs, including internet, private spaces, financial supports, childcare, and adequate food – the latter being a particular point of tension for participants. Many women experiencing homelessness would not have had the financial means to provide what the shelter could not (as the above participant, V73, had), compounding stress. In some cases, shelter rules created additional challenges for VAW participants caring for their children. For instance, regarding her stay in a VAW shelter, one participant (V74, white woman, single mother) shared:

The tour and the intake process took about an hour, an hour and a half. My daughter and I were separated and that was really hard because we had no say in the matter. So, right away, it was not a, “You’re safe here.” […] And I know my daughter is freaking out, not because she’s screaming, but I just know her. And I’m like, “Can I just go and make sure that she’s okay?” […] And then we just sat in the office. They left us alone and we just cried for 45 min. […] They told us that we could not sleep together. That [my daughter] had to sleep on one wall. I had to sleep on another. I thought that that was very interesting. So, it was for fire safety code. And then we finally fell asleep […] and then somebody banged on the door at 10 o’clock. […] She comes right in the door and she says, “You have to get up.” And I and [my daughter] starts screaming […] And she’s like, “Why are you asleep?” And because we’re asleep, “Because you’re not supposed to be sleeping together […] She totally was like, “Oh, I’m sorry, I’m just wondering if you have any drugs? You need to bring them to the front office.” And I’m like, “Excuse me?” She’s like, “Do you have any drugs?”

This participant’s story demonstrates a common experience among several VAW participants where a lack of a trauma-informed or child-centred lens in the implementation of shelter rules was experienced as retraumatizing for those caring for children. In addition, the excerpt illustrates the common abstinence-only approach applied in many VAW shelters, which not only was a jarring experience for the participant to be questioned about in front of her daughter but generally acted as an access barrier for women experiencing violence and homelessness who also use substances (as was the case for most encampment participants).

A lack of child-specific programming or support in general was a major limitation in the shelter system experienced by VAW participants, which often resulted in women having little to no respite from caregiving (e.g., as exemplified by participant V78 above). This was particularly challenging when confined to a room during the COVID-19 pandemic, without being allowed to move around the building or go outside. An extreme case was experienced by one VAW participant (V77, South Asian woman, newcomer), when she was admitted to hospital and VAW shelter staff contacted child protection services for the participant’s daughter due to a lack of childcare onsite – a distressing and disempowering experience. In contrast, the availability of child supports was often central to participants’ interpretation of their shelter experiences as positive. For instance, one participant (V79, East Asian woman, newcomer) described, “[The VAW shelter] was really good, it was supportive. […] They provided interpretation service and also my kids’ food and clothing. […] There were children’s counselors in the shelter and then I could discuss about my children’s behavior and then they helped me a lot.” As this participant demonstrates, physical resources as well as mental health supports for children in shelter were critical for family well-being.

#### Gender transformative support

Experiences of shelter were situational to the environment and supports in place, depending on whether they promoted or impeded upon women’s empowerment. This was evident in participants’ juxtaposing experiences when moving from one shelter to another. In some cases, participants’ shift from a negative to positive shelter experience coincided with the availability of gender-specific support and programming. For instance, following her negative experience in an emergency shelter, a VAW participant (V73) described her time in a VAW shelter with her two children living with autism:

They talked to you like a human. We make jokes, we laugh. If they have anything funny to share, they’ll talk to us. They didn’t make you feel broken and that was the best part. Like I have one of the staff who was amazingly great with me and she used to work with disability kids. And like, if I’m having a hard time with the girls. I will cry to her and I’m like, I can’t do this right now. Like, they’re having a huge meltdown and she’ll tell me, she teach me some ways how to deal […] I have so much love for them, for them who helped me grow as a single mother of two disability children, cause that I felt like at the beginning and never could’ve did it by myself.

As this participant illustrates, staff who treated residents with dignity, without judgment, and fostered social connection, created a shelter environment in which residents felt empowered and could advance in their healing journeys. Another participant (V77) compared her transformative experience in one VAW shelter to a traumatic experience in another, sharing, “That was the big thing for me, that they were behaving with my child like family.” As these quotes demonstrate, critical to a gender-transformative approach for most VAW participants was also the provision of appropriate parenting and child-based supports. As exemplified above, when even gender-specific shelters, including VAW shelters, did not have these supports, it led to harmful experiences for VAW participants (e.g., participants V73, V74, V77, V78). Participant V72 (white woman, age 42) explicitly connected the empathic approaches that participants V73 and V77 described to redressing gender inequities:

People feel that vibe that you’re bringing to the table when you don’t, when you have like a disdain or when you don’t really feel that they should be getting the help or they should be getting the support – people feel that. And I think seriously looking and being so intentional in training people for those roles within the VAW and against gender-based violence is so critically important because [sigh] you know, you just, you have so many women, unfortunately, and female-identified individuals that are going through so much pain and they just need some, like something or some kind of compassion or empathy and to know there’s a different way.

This quote makes clear yet again how gender stereotypes around women’s deservingness embedded at the organizational level can stigmatize women and deter their progress. Implementing a gender-transformative approach to homelessness and VAW requires investment across systems to not only enhance capacity, staffing, and programming, but also significant training around implicit gender-based and other discriminatory biases and the provision of trauma-informed care.

## Discussion

This study uniquely combined first voice data among participants from the VAW and homelessness sectors to critically interrogate the social construction of ‘homelessness’ and the power relations that dictate *who* and *how* different women access different support systems. We found that participants across the VAW sector and encampments commonly experienced traditional pathways into women’s homelessness as in pre-pandemic times – namely those centred around experiences of VAW and the gendered abuse of power and control [42, 43]. However, the COVID-19 pandemic presented new opportunities for people (especially men) to leverage power and control over participants (e.g., using public health measures to isolate survivors further), while exacerbating capacity limitations at supportive organizations, including fewer available shelter spaces, greater concerns over safety, and more limited wraparound support. This was most acutely felt by participants who were facing overlapping forms of marginalization, such as economic or immigration precarity. The common pathways into women’s homelessness across support systems highlights the importance of adopting more inclusive definitions to homelessness and policy targets that take into account these experiences – including defining living in violent, abusive, or precarious environments as experiencing homelessness [15].

Across the VAW and homelessness sectors, constructs of safety and autonomy were central to shaping experiences of women’s homelessness. The shelter system – and especially the homeless shelter system – was often perceived and experienced as a space that threatened both autonomy and safety due to limitations in the built and social environments. This aligns with past literature around shelter systems, especially mixed-gender shelters, as places antithetical to individual autonomy and mutual support, where concerns of surveillance are prevalent, and which do not consistently meet the needs of the diversity of people who may access them (e.g., gender-blind approaches that may lead to women experiencing further male violence; abstinence-only models that do not support the needs of people using drugs; or physical environments that are inaccessible to people living with disabilities) [12, 13, 44].

Encampments often stood in contrast to the homeless shelter system, as places that participants felt promoted their autonomy and where residents could offer each other mutual support [27]. However, participants across gender identities commonly acknowledged the additional safety concerns for women within encampments, especially experiences of sexual violence. Women thus faced significant challenges to their safety across many (if not, at times, all) of their available options – from staying in or leaving abusive situations to homeless shelters or encampments. Specific to encampments, participants described how they negotiated safety (typically by drawing on male protection, or at times establishing ‘mothering’ roles for themselves) and in the process strengthened social bonds and, as one participant put it, realized “that humanity really does care about each other.” This reflects the critical importance of gender-transformative approaches to housing that promote both safety and empowerment [45, 46].

Relatedly, participants described the ways in which gender stereotypes, both internalized and at a structural or organizational level, harmed them in terms of the services or supports they accessed. Similar to earlier research with women accessing homeless services in Ireland [35], participants were affected by a social discourse around *which* women were deserving of formalized support (i.e., those closest to fitting the stereotype of the ‘ideal woman’), which included those with more education, seeking upward mobility, not on social assistance, or not using alcohol or other substances. In general, women were viewed as needing to be productive in the eyes of society to be deserving of any support; women needing more support or resources than ‘typical’ (e.g., due to poverty, disability, immigration status, culture, or lone caregiving) were seen as a societal burden. This may, in part, reflect a neoliberal discourse in which welfare has shifted to ‘workfare,’ whereby recipients of social assistance are expected to contribute to the workforce [47]. Critically, our findings highlighted the ways in which this discourse led to women who use drugs exclusively accessing supports within the housing and homelessness sector rather than VAW sector, due to the typical abstinence-only approach of VAW organizations. Our results demonstrate how the absence of an intersectional approach to supportive programming and policy across housing, homelessness, and VAW further harms and marginalizes women [48].

The most positive experiences that participants described when accessing supportive services were when they received gender- and VAW-specific supports that promoted their safety and autonomy – in many cases, despite the constraints presented by COVID-19-related public health measures. Parenting in particular represented an important area for participants with children to feel well supported in and, when they were not, negatively impacted wellbeing. This discussion was entirely centred within the MARCO-VAW dataset, which likely reflects the limited child and family strengthening support available within the homelessness system [49, 50]. Our findings extend prior research that has shown that the presence of children facilitates access to housing [35]. While we similarly found that the living options women accessed differed based on the presence of children, in many ways, women who were lone caregivers to their children were given little choice around how they could exit violent situations if they were to maintain custody. Often, they were forced to take the first available shelter option, regardless of safety or availability of parenting or child supports, with most experiencing significant harm and revictimization as a result (including risk of child apprehension). Single caregiver families are most often female-led, making parenting in the context of VAW and homelessness an essential consideration for gender-transformative approaches to homelessness across the VAW and housing and homelessness sectors (i.e., including wraparound, trauma-informed child programming and parenting supports) [12]. The lack of discussion amongst encampment participants of parenting or childcare needs (a greater proportion of whom used drugs and were Indigenous), further highlights the call to action of previous Canadian research to consider the ways in which certain women are further structurally disadvantaged from maintaining child custody [12]. This has implications for women’s perceived deservingness of housing or VAW supports, and ultimately the perpetuation of cycles of homelessness and violence.

### Strengths and limitations

This study contributes to the literature by combining firsthand data on experiences of women’s homelessness from across the VAW and homelessness systems. Our community-based research relied on strong partnerships with knowledge users and people with lived experience of violence and homelessness as research team members and advisors. We maintained active engagement with our partners and collaborators throughout the study using integrated knowledge translation, ensuring that the project reflected the realities being experienced on the ground. Our sample was high in “informational power” for our research questions, providing rich and detailed accounts that allowed us to generate a nuanced analysis [51]. We were committed to capturing the stories of participants from a diversity of personal and social identities and especially those experiencing different forms of marginalization. Our analysis generated a number of different learnings (and, subsequently, directions for systems and social change) around the ways in which structural disadvantage and oppression affected women differently based on overlapping factors such as gender, substance use, caregiving status, immigration status, and disability, to name a few. Future studies could build on this work by examining women’s experiences of homelessness and violence specific to different communities, which may produce further insights around intersectional experiences involving race and culture, in particular. This should include, for instance, Indigenous women, who are at greater risk for experiencing violence and homelessness, especially in light of the calls to justice from the National Inquiry into Missing and Murdered Indigenous Women and Girls, including for Indigenous-led housing and homeless services [52].

Our study represents a nuanced analysis of experiences of violence, homelessness, and service access at the height of the COVID-19 pandemic (2020–2021) in the Greater Toronto Area. Toronto is Canada’s largest and most diverse city, which, in comparison to other Canadian cities, is better resourced in terms of health, social, and community services. This makes it a particularly important context nationally for informing housing policy and coordinated action across systems, especially to meet the needs of women facing multiple forms of marginalization. However, future research is needed to investigate potential differences and unique needs within other municipalities (including rural or remote areas), in Canada and internationally. In addition, while the MARCO-VAW and MARCO-Encampments studies were conducted with similar methodological approaches, with the study leads in communication throughout, data were ultimately collected by separate teams. Violence and gender were not part of the central research questions of the MARCO-Encampments study, despite being present in the data, which means there are areas where richer data may be obtained in future studies. Likewise, while gender expansive and sexual minority people were eligible participants in both studies, most MARCO-VAW study participants identified as heterosexual, cisgender women – reflective of the characteristics of the majority of VAW organizational clientele and potential service access barriers [53]. In addition, most discussion around gender-based violence and gender-based homelessness in the MARCO-Encampments study came from cis women participants. Therefore, a critical area for future research is purposeful sampling of gender and sexual minority participants to better understand potential similarities and differences in experiences of homelessness, violence, and barriers, facilitators, and outcomes of related service access across sectors. However, (albeit based on limited data), the commonalities we observed across cis women and gender expansive participants speaks to the continued importance of an inclusive approach to gender transformative programming across sectors (including the VAW sector).

## Conclusions

The traditional cycle of VAW leading to and resulting from women’s homelessness was exacerbated by the COVID-19 pandemic, especially for those women facing multiple forms of marginalization, like precarious migrant status, poverty, disability, and substance use issues. In navigating this cycle, women faced complex decisions that involved negotiating trade-offs between safety versus autonomy. Gender- and VAW-specific supports had the greatest potential of maximizing safety and promoting empowerment, but, in addition to capacity limitations, gender stereotypes and multiple marginalization served as a structural barrier to access.

Our results highlight the need for strengthened intersectoral coordination between VAW and homelessness sectors and a unified policy strategy to addressing homelessness that applies a gender-transformative and intersectional approach. This should include adopting a standardized and inclusive definition of homelessness that accounts for women’s hidden homelessness to inform homelessness policy and programmatic targets, eligibility criteria, and funding. Sustainable investment should be made to enhance capacity, staffing, programming, and training across VAW and homeless systems to deliver gender-specific, trauma-informed services, including emergency shelter, that promote *all* clients’ autonomy and safety, including those with complex needs (e.g., incorporating harm reduction approaches). Trauma-informed and equitable service provision must include attention paid to the built environment, both in terms of accessibility and safety concerns for women. Supportive services for women’s homelessness and VAW should offer parenting and child support, in recognition of the disproportionate caregiving burden among women. Appropriate funding and resourcing must also be provided to VAW and homelessness organizations to develop public health emergency preparedness to ensure that these considerations are integrated into infection prevention and control protocols. Finally, greater investment in affordable housing is needed, but initiatives must also take into account safety and accessibility, while rapid rehousing programs for women should be coupled with wraparound VAW supports.

## Electronic supplementary material

Below is the link to the electronic supplementary material.

Supplementary Material 1

## Data Availability

Data are not available for sharing due to their sensitive nature and the risk for identification.

## Abbreviations

VAW: Violence against women
