# Supplementary material for: Women’s experiences of homelessness and violence during the COVID-19 pandemic in Canada’s largest city: an integrated qualitative analysis of clients of violence against women organizations and encampment residents
Source: BMC Public Health. 2025 Apr 28;25:1529. doi: 10.1186/s12889-025-22721-4 (PMC12036223; doi:10.1186/s12889-025-22721-4)
Supplement: Supplementary file 1 — Supplementary Material 1 [file 12889_2025_22721_MOESM1_ESM.pdf]

Additional file 1: MARCO-VAW and MARCO-Encampments Interview Guides

A) MARCO-VAW Study: Semi-Structured Guide for Survivor Interviews

> ***Note for researchers:*** *We will not necessarily say everything below verbatim – this is just a guide to help the interview proceed smoothly. As each participant shares their experiences, we will ask follow-up questions based on what they said and adapt the questions below as needed.* ***Italics are used below to denote prompts, which are example follow-up questions, and may not be discussed with every participant.***

Introduction

Thank you so much for taking the time to speak with me today. We want to hear about your experiences with violence against women or VAW services during the COVID-19 pandemic. Our goal is to draw actionable recommendations to strengthen the VAW response in this and future public health emergencies.

Before we begin, do you feel you are in a comfortable and private space to talk? [If yes, proceed. If not, is there somewhere else you can speak to me or is there a better time we can reschedule to?] If at any point you need to stop or pause the interview and pick up at another time, please don't hesitate to let me know.

As a reminder, this interview will last about 1 to 1.5 hours. You do not have to answer all of the questions and, again, we can pause or stop the interview at any time, for any reason. With your consent we will be recording our conversation. The audio recording of this interview will be securely destroyed after it is recorded in writing. All information will be kept confidential and any identifying information you share about yourself or others (like names) will be removed from the final data file.

Do you have any questions before we get started?

[Start recording]

Topic 1: About you

1. To start, would you be able to tell me a little bit about your day to day?

[Prompts, if needed]

- *Working outside the home? Working from home?*
- *Any children? Ages? Do they live with you? Are they in school? Virtual school?*

2. [For non-shelter participants]: We're interested in understanding how representative our interviews are and how far our participants are going to access services. Can you tell me what part of Toronto you live in?
    - *Would you feel comfortable telling me about your living situation?*

    [For shelter participants]: Would you feel comfortable telling me about your living situation?
    - *We're interested in understanding how representative our interviews are and how far our participants are going to access services. Can you tell me a little bit about where you lived prior to now? (e.g., were you in Toronto/different city? What did your living situation look like?)*

Topic 2: Impact of the pandemic

3. [De-prioritize if participant has given a lot of related information]. How has your life changed during the pandemic?
    - *Day-to-day? Work? Childcare?*
    - *Relationships? Social life?*
    - *Economic situation?*
    - *Mental health? Physical health?*

Topic 3: Experience of violence

4. Can you tell me about your most recent relationship where there was violence or abuse?
    - *Nature of the relationship (family member or dating, living together, married, something else)*
    - *Status of the relationship (e.g., partner or ex-partner; still in communication or not)*

5. Did the relationship begin before or during the pandemic?
    - *When did the abuse first begin?*

a) [Wording depends on when the relationship began]: In what ways, if any, did the relationship change during the pandemic (compared to before) or over the course of the pandemic?
    - *Were there ways in which the nature of the abuse or violence changed?*
    - *How did pandemic restrictions or fear around COVID-19 impact the relationship?*
    - *What, if any, new situations during the pandemic caused stress in the relationship, like children at home, partner at home, loss of tenancy, loss of work?*
    - *What does the contact between the both of you look like now? In what, if any, ways did the pandemic impact this?*

Topic 4: Service access and outcomes

6. Related to the abuse or violence you experienced, where did you seek help in Toronto?
    - *Name(s) of organization(s)*
    - *What kinds of support did you receive (e.g., crisis hotline, counselling, shelter, legal support, housing supports)?*
    - *Virtual? In person?*
    - *Residential or non-residential?*

a) How did you decide where to seek services?
    - *Found online? Referred (if so, by who)?*
    - *What motivated you to connect with services (e.g., friends or family)?*
    - *Was there a particular approach or program that impacted where you sought services (e.g., peer support, trauma-informed, specific language services, specific community orientation)?*
    - *Was this an/were these organization(s) you had previously reached out to?*
    - *What has navigating where to find support from services or organizations in Toronto been like during the pandemic? (How does this compare to before, if relevant?)*

7. What were your experiences like in accessing these services?
    - *How helpful did you find the services you accessed? In what ways were these helpful or not helpful?*
    - *Did you access services before COVID as well? If so, how has the pandemic changed your experience?*

a) What, if any, were the greatest challenges in accessing VAW services during the pandemic?
b) How concerned were you about contracting COVID-19? In what ways, if any, did this impact how you engaged with VAW services?
c) [If participant accessed in person services]: What was your experience with PPE like?
    - What did you wear? Who provided it? Were there any ways this impacted your experience?
d) [If participant accessed residential services]: Did you stay in a hotel as part of your shelter stay? If so, can you tell me about that experience?
    - What was the transition into shelter like? (For you? For your kids, if relevant?)
    - [If relevant]: What was the transition out of shelter like? (For you? For your kids, if relevant?)
e) [If participant accessed virtual services]: What was it like accessing services virtually?
    - Did you experience any challenges with technology (if so, what)? (E.g., did you have difficulties accessing a phone, computer, or internet, or challenges with privacy?)
    - What benefits, if any, have there been to virtual services?

8. How supported did you feel by the organization(s)?
    - *In what ways, if any, did you feel supported by the organization(s)? Do you feel your kids (if relevant) were supported?*
    - *In what ways, if any, could the organization have better supported you? Your kids, if relevant?*
    - *What would your ideal experience look like? What do you wish would be done for VAW survivors in Toronto?*

9. Where do you feel you are at now in your healing journey?
    - *How is your mental health? How is your physical health? What do your relationships look like?*
    - *Where are you hoping to get to? What does getting there look like for you?*
    - *In what ways, if any, has the pandemic impacted this?*

Topic 5: Contextual factors

We are on the last section and I only have a few more questions.

10. During the pandemic, were there other issues happening in the world or your community that impacted you on a personal level (e.g., Black Lives Matter, COVID-19 related protests, anti-Asian racism, anti-Black racism, politics, recession)?
    - *If yes, can you tell me about your experiences?*
    - *Were there any times you felt discriminated against during the pandemic?*
    - *What ways, if any, did these experiences intersect with your experience of violence? Or your experience accessing VAW services?*

11. Canada is currently investing $600 million over 5 years to prevent gender-based violence under the National Action Plan to End Gender-Based Violence. This includes funds *to enhance the capacity of VAW organizations, Indigenous women organizations, and crisis hotline.* What do you think would be helpful to include in this plan?
    - *What immediate changes should be made in Canada to better respond to VAW?*
    - *What long-term changes should be made to prevent VAW from happening in the first place?*

Closing

12. Thank you for answering my questions. We are at the end of the interview and we've talked about difficult things today. How has talking about these things made you feel?

13. Those are all of my questions. Is there anything else you feel is important to know about your experience of accessing VAW services during the pandemic?

Thank you for participating in today's interview. We appreciate your time and your lived expertise on these issues. We hope that with information like what you have shared today we will be able to make a difference in the lives of survivors during and beyond the pandemic.

[End recording]

Thank you so much. Our research coordinator, Kimia, will follow up with you over email to coordinate your honorarium and any opportunities to participate in our study or be informed about our findings.

B) MARCO-Encampment Study: Semi-Structured Guide for Resident Interviews

**Note for researchers:** *We will not necessarily say everything below verbatim – this is just a guide to help the interview proceed smoothly. As each participant shares their experiences, we will ask follow-up questions based on what they said and adapt the questions below as needed.* ***Italics are used below to denote prompts, which are example follow-up questions, and may not be discussed with every participant.***

Introduction

Thank you so much for taking the time to speak with me today. We want to hear about your experiences and involvement with the encampments in Toronto. I would like to remind you that this interview will be recorded. All information will be kept confidential and any information you share about your personal identity (or the personal identities of others) will be removed from the final data file. The recording will be securely destroyed after being transferred into a written document and safely stored.

Before we get started, do you have any questions? [turn on recorder after this]

Topic 1: COVID-19-related homelessness and encampment living

1. To start, can you please tell me a bit about what happened that led you to move to an encampment during the pandemic?
    - *Where were you staying before? What was it like? Why did you leave? How did this influence your decision to move to the encampment?*
    - *Did the pandemic influence your decision to move to an encampment? In what ways?*
    - *Did you have any other options? Why did you choose an encampment over them?*

2. What were your expectations about the encampments before you moved in?
    - *What had you heard about the encampments before you moved there?*

- *Were you already connected with anyone who was staying in an encampment? How did you know that joining an encampment was an option for you?*
- *Did you hear anything about whether or not the city was offering shelter to encampment residents before you moved in?*
- *How long were you thinking that you would stay?*

3. What was your overall experience like staying in the encampment?
   - *Can you tell me a bit about your day-to-day routine in the encampment? How was it the same or different from before you moved to the encampment?*
   - *What was your relationship like with other residents?*
   - *How did the residents of the encampment share the space on a day-to-day basis? Did you all work together, or mainly fend for yourselves? Was there any kind of leadership or decision making structures? How did those work? Were there any rules for staying in the encampment? What were they?*
   - *Did you have a particular role within the encampment?*
   - *What was your experience of the people that were passing through the encampment area? (e.g. community members, neighbours, police, outreach) How did interactions with these people affect you?*

4. How would you describe your feelings of safety or security while staying in the encampment?
   - What kinds of things made you feel safe? What made you feel unsafe?
   - Did you experience any stigma, harassment, or acts of violence while in the encampment? *[If yes] Are you open to sharing what happened? What was that like?*
     - *Who was involved (e.g. neighbours, city staff, police)?*
     - *Were these kinds of situations common? How did you handle them?*
     - Do you feel that any of these experiences were related to any specific form of prejudice (e.g. racism, sexism, homophobia, transphobia, poverty, drug use, ableism)?

Topic 2: Encampment outreach supports

5. Tell me about the outreach supports you received while staying in an encampment – what do you think of them overall?
   - *Which groups of workers/volunteers did you receive help from (if you can identify them – e.g. Streets to Homes, South Riverdale CHC, Sanctuary, Anishinawbe Health, ESN, ALAB Legal Info Clinic, Parkdale Queen West CHC)?*
     - *Which groups were most/least helpful and why?*
   - *Which supports did you like or helped you the most? Why?*
   - *Which supports did you not like or helped you the least? Why?*

6. How did the outreach supports you received in the encampment help you to meet your needs?
   - *[e.g. Consider asking about each of the following basic needs:] water, hygiene/sanitation, fire safety, waste management, health/social supports, personal safety, food safety, harm reduction, pest/rodent prevention, resources to protect against seasonal issues*
   - How did the outreach supports influence your feeling of personal security (e.g. in the face of violence, harassment, or stigma)?
   - *What kind of information did you receive from outreach support workers/volunteers? How did it help you or not help you?*
   - Aside from the outreach supports, did you receive any support from other people in your life (e.g. family/friends)? [If yes] What kind of supports? How did they help you to meet your needs?

7. Were you using any substances during your stay in an encampment?
   - [If yes] What was it like using in the encampment? How does this compare to other shelter/housing you have had during the pandemic?
   - How do you feel your risk related to using substances was affected by staying in an encampment (e.g. overdose risk)? How does this compare to other shelter/housing you have had/may have during the pandemic?
   - What was your experience with accessing any harm reduction supports you needed from encampment outreach workers? How did this affect your substance use and sense of safety in the encampment?
   - Have you experienced or witnessed an overdose during the pandemic? Where were you? What supports were available in this situation (if any)? How were they helpful?

8. Did you have any immediate medical needs while staying in an encampment *(e.g. abscesses, other wounds, acute psychosis)*? [If yes] How did you access the care you needed?
   - Did the outreach supports help you to address these? How so?
   - Were you accessing any healthcare for any long-term health issues you have while you were in the encampment *(e.g. mental or physical health conditions, chronic pain, HIV)*?
     - Did the outreach supports help you to care for these? How so?

9. Are there any types of healthcare that you feel you need but haven't been able to access since moving to an encampment? Like what?
   - *What are the barriers to accessing the care you need?*

10. In what ways did the encampment outreach supports/workers respect or not respect your personal autonomy? *(i.e. feeling that you are willingly engaging in your behaviours, rather*

than feeling pressured to take certain actions)

- How does this compare to other shelter/housing you have stayed in?

11. How did the outreach supports/workers in the encampment affect your connection to the community? *(i.e. feeling of belonging or inclusion)*
    - How does this compare to other shelter/housing you have stayed in?

12. Which of your needs have not been met while staying in an encampment? How so?
    - *Did you find any of the supports affected you in a negative way? How so?*
        - *How could the supports you receive, or the way you receive them, in an encampment be improved?*
    - *Are there any other supports that you did not receive that would have been helpful to you in an encampment?*
    - *What do you anticipate will be most challenging for you going forward? What do you think is needed to address this?*

Topic 3: Encampment housing alternatives and displacement

13. Were you ever offered any alternative shelter/housing options during the pandemic? *(e.g. traditional shelter bed, shelter hotel, temporary or permanent housing)*
    - Can you tell me about how this happened? Who offered them? Were any offered while you were staying in an encampment? [If yes] Did you accept? Why or why not?
        - *[If did not accept, consider asking about the following concerns:] COVID-19 risk, overdose risk, other health risks, unsanitary conditions, crowding, rigid/controlling rules or restrictions, stigma or harassment or violence, theft of belongings, other negative treatment, isolation, distant from community/support system*
        - [If accepted] What happened after you accepted?
            - *What information did you receive about it in advance? What was the moving process like? What did you think of it once staying there? How long did you stay?*
            - Did you later return to stay in an encampment again? Why? *[If returned to encampment, consider asking about the following concerns:] COVID-19 risk, overdose risk, other health risks, unsanitary conditions, crowding, rigid/controlling rules or restrictions, stigma or harassment or violence, theft of belongings, other negative treatment, isolation, distant from community/support system*

14. Did you experience an eviction while staying in an encampment? [If yes] How did it happen?
    - *Who was involved? How were you treated during the process?*
    - *Was it after you were offered alternative shelter/housing?*
    - *What information or notice did you receive in advance (if any)?*

- *What did you do after being evicted?*

15. What does housing that meets your needs and is accessible to you look like?
    - *Has the pandemic influenced your specific housing needs? In what ways?*
    - *What are the key barriers to accessing the kind of housing that you need?*
    - *Did any of the outreach supports you received while staying in an encampment help you overcome any of these barriers?*
        - *[If stayed in other shelter/housing during the pandemic] Did any of the supports you received while staying in [other shelter/housing option] help you to access the housing you need?*

Topic 4: COVID-19-related supports

16. How do you think your risk of getting COVID-19 in an encampment compares to the risk in other shelter/housing options you have stayed in or been offered?
    - *How concerned are you about getting COVID-19? Why is that?*

17. What kinds of things have you been doing to reduce your risk of getting COVID-19 (if any)? *(e.g. mask, hand washing/sanitizer, physical distancing, staying in encampment)*
    - Did the outreach supports you received in an encampment help you prevent COVID-19 transmission? How so?
        - *What kinds of COVID-19-related services and supplies did you receive in the encampment? What about while you were staying in any other shelter/housing?*
        - *How did you feel about these supports? How did they affect your experience of the pandemic?*

18. Did you ever access the mobile COVID-19 testing service?
    - *[If yes] What was your experience with it? If you were also tested elsewhere, how did that experience compare?*

19. Did you ever test positive for COVID-19 while staying in an encampment?
    - *[If yes] What was your experience with COVID-19? Where do you think you got it from? Where did you go when you tested positive? [If left encampment] How did this compare to the encampment (especially in terms of supports you had access to)?*

Questions to wrap up interview

20. Have you been involved in any shelter or housing-related advocacy in Toronto?
    - *[If yes] What kinds of things have you advocated for? (e.g. improvements to living conditions within the support options available, ending encampment evictions, affordable*

housing)

- *[If yes] How did you become involved in this advocacy?*
    - *Is this connected to the outreach supports you received in an encampment? Is this a change from before the pandemic or before you moved to an encampment?*
- *How do you feel about the advocacy that has been happening to support the encampments during the pandemic?*
    - *How do you think this advocacy has affected your experience of the encampments?*

21. Is there anything else about encampments/housing/outreach supports that is important to you that we did not discuss enough during this interview?
    - *Anything you thought I would ask you but didn't?*

This concludes today's interview. Thank you for sharing your knowledge and experiences, we are very grateful.

[END INTERVIEW]
